# Isolation and partial chemical characterization of macrophage-derived neutrophil chemotactic factor

**DOI:** 10.1155/S096293519500041X

**Published:** 1995-07

**Authors:** M. Dias-Baruffi, M. C. Roque-Barreira, F. Q. Cunha, S. H. Ferreira

**Affiliations:** 1 Department of Immunology Faculty of Medicine São Paulo, 14 Ribeirão Preto 049-900 Brazil; 2 Department of pharmacology Faculty of Medicine São Paulo, 14 Ribeirão Preto 049-900 Brazil

## Abstract

Macrophages stimulated with lipopolysaccharide (LPS) release a factor (MNCF; macrophage-derived neutrophil chemotactic factor) which induces neutrophil migration *in vivo* and *in vitro*. The *in vivo* chemotactic activity of crude MNCF is not affected by pretreating the animals with dexamethasone, an uncommon characteristic which discriminates MNCF from known chemotactic cytokines. We purified MNCF by affinity chromatography of the supernatant from LPS-stimulated macrophages on immobilized D-galactose, followed by gel filtration of the sugar-binding material on Superdex 75. The activity was eluted in the volume corresponding to a MW of 54 kDa. SDS–PAGE of this preparation revealed a single band, also corresponding to a 54 kDa protein. MNCF is an acidic protein (pI < 4) as shown by chromatofocussing. Like the crude MNCF, the homogeneous protein induced neutrophil migration *in vitro* as well as *in vivo*. This was not modified by dexamethasone pretreatment.


Mediators of Inflammation 4, 257–262 (1995)

### Isolation and partial chemical characterization of macrophage-derived neutrophil chemotactic factor

M. Dias-Baruffi,[1] M. C. Roque-Barreira,[1,CA] F. Q. Cunha[2] and S. H. Ferreira[2]

Departments of [1]Immunology and [2]Pharmacology, Faculty of Medicine, Ribeirão Preto, São Paulo, 14,049-900, Brazil

[CA]Corresponding Author

**MACROPHAGES stimulated with lipopolysaccharide (LPS) release a factor (MNCF; macrophage-derived neutrophil chemotactic factor) which induces neutrophil migration *in vivo* and *in vitro*. The *in vivo* chemotactic activity of crude MNCF is not affected by pretreating the animals with dexamethasone, an uncommon characteristic which discriminates MNCF from known chemotactic cytokines. We purified MNCF by affinity chromatography of the supernatant from LPS-stimulated macrophages on immobilized D-galactose, followed by gel filtration of the sugar-binding material on Superdex 75. The activity was eluted in the volume corresponding to a MW of 54 kDa. SDS–PAGE of this preparation revealed a single band, also corresponding to a 54 kDa protein. MNCF is an acidic protein (pI < 4) as shown by chromatofocussing. Like the crude MNCF, the homogeneous protein induced neutrophil migration *in vitro* as well as *in vivo*. This was not modified by dexamethasone pretreatment.**

**Key words:** Inflammation, Leukocyte, Monokine

### Introduction

Resident macrophages act as alarm cells in the inflammatory process by virtue of their rapid and intense secretory response to exogenous and/or endogenous activating stimuli.[1–4] More than 100 products released by macrophages activated by various types of stimuli have been described.[4,5] Among them are cytokines, products of arachidonic acid and components of the complement system, many with demonstrated inflammatory activities. Interleukin 1 and 8 (IL-1, IL-8), tumour necrosis factor (TNF), the complement fragments and leukotriene $B_4$ ($LTB_4$) are macrophage products thought to be involved in the migration of neutrophils to the inflamed site, a phenomenon characteristic of the acute phase of the inflammatory process.[4,6–8] Previous studies from our laboratory have demonstrated that activated macrophages release a chemotactic factor for neutrophils (macrophage-derived neutrophil chemotactic factor, MNCF) which is active when tested *in vitro* and in the peritoneal cavity of rats. Neutrophil migration into the abdominal cavity induced by MNCF is not reduced in animals pretreated with dexamethasone or by peritoneal cell depletion. Thus, the chemotactic activity of this factor seems to be independent of resident cells. Among the cytokines capable of inducing neutrophil migration, only chemokines such as IL-8 have a direct chemotactic action since they are active when tested in a Boyden chamber. However, we have observed that the *in vivo* migration induced by IL-8 is blocked by pretreating the animals with glucocorticoids and appears to be dependent on mast cells.[9] The chemotactic dependence of IL-8 on mast cells was confirmed by others.[10] These biological characteristics, which differ from those of MNCF, led us to suggest that MNCF does not correspond to any of the monokines described in the literature.[9,11,12]

We thus consider the induction of neutrophil migration in rats pre-treated with glucocorticoids to be peculiar to MNCF. In the present study, we describe the two-step purification of MNCF from the supernatant of rat peritoneal macrophage monolayers stimulated with LPS. We also describe some chemical characteristics of MNCF.

### Materials and Methods

*Animals:* Male, albino, Wistar rats (*Rattus norvegicus*) weighing from 180 to 200 g and maintained in temperature-controlled rooms at 23–25°C with free access to food and water were used as the source of peritoneal macrophages as well as for the *in vivo* tests of cell migration.

*Production of neutrophil chemotactic factor by LPS-stimulated macrophages:* MNCF was produced under aseptic conditions as described by

© 1995 Rapid Science Publishers

Cunha and Ferreira.[12] Briefly, macrophages were obtained from the peritoneal cavity of rats pretreated with thioglycollate (10 ml of a 3% solution, injected intraperitoneally 4 days before the experiments). To harvest the thioglycollate-elicited macrophages, the rats were sacrificed by cervical dislocation and immediately injected intraperitoneally with 10 ml of heparinized RPMI 1640 (5 IU/ml). Each peritoneal wash was aspirated and placed in a separate plastic Petri dish; the dishes were subsequently incubated at 37°C for 60 min in a $CO_2$ incubator. The supernatants were discarded and the adhering cells were gently washed twice with PBS to eliminate cell debris as well as any cells which had not adhered to the plastic surface. The macrophage monolayers were incubated with LPS diluted in RPMI 1640 (5 μg/ml medium) for 30 min, after which time the solution containing LPS was discarded and the monolayers were gently washed three times with PBS. The cells were then incubated for a further 90 min with RPMI 1640 in the absence of LPS. These are ideal conditions for the maximal release of MNCF into the supernatant.[12]

Cell counts and analysis of mononuclear cell viability were performed in 5% of all the monolayers used. The adhering cells were suspended in 1 ml of heparinized RPMI 1640, submitted to total and differential counts and analysed for viability based on the technique of eosin Y exclusion. Throughout the experiments, each plate contained an average of $3.6 \pm 0.6 \times 10^6$ viable macrophages.

The crude supernatant solutions (150 to 250 ml) were centrifuged (2 000 × **g** for 5 min at 25°C) and ultradiafiltered through YM-10 Amicon membranes against sterile, deionized water at 4°C. They were then concentrated to 1 or 2 ml, sterilized by filtration through 0.22 μm pore filters (Millipore Corporation, Bedford, MA, USA) and either stored at −70°C or maintained at 4°C in an ice bath in a cold chamber when they were to be used immediately. This preparation was designated as crude MNCF.

*Chromatographic procedures for the fractionation of crude MNCF:* The chromatographic separation of MNCF was monitored by measuring the effluent absorbance at 280 and/or 230 nm using a DU-70 spectrophotometer (Beckman Instruments, Fullerton, CA, USA) or a UV-MII detector (Pharmacia LKB Biotechnology, Uppsala, Sweden), and by measuring the biological activity.

*Affinity on immobilized sugar resins:* Crude MNCF derived from $3.6 \times 10^8$ cells was chromatographed at 4°C on agarose/D-galactose columns (Pierce Chemical Co., Rockford, IL, USA). The material not retained by the column was eluted with sterile, deionized water, and the retarded substances were eluted with 0.4 M D-galactose. The two fractions (D-gal− and D-gal+) were ultradiafiltered through YM-10 membranes (Amicon Division, W.R. Grace & Co., Beverly, MA, USA) against sterile, deionized water at 4°C. A similar procedure was performed on an agarose/D-mannose column and the sugar-bound fraction was eluted with 0.4 M D-mannose.

*Gel filtration on Superdex® 75:* The D-gal+ binding fraction obtained by affinity chromatography was concentrated to 0.2 ml and submitted to gel filtration on a Superdex® 75 HR 10/30 column (Pharmacia LKB Biotechnology, Uppsala, Sweden) equilibrated with 10 mM PBS, pH 7.4, and operated at 20°C with a flow rate of 0.5 ml/min. Fractions of 0.5 ml each were collected. The column was previously standardized with known molecular weight markers, i.e., blue dextran (2 000 kDa), phosphorylase B (94 kDa), bovine serum albumin (BSA, 67 kDa), chicken egg albumin (43 kDa), chymotrypsinogen (25 kDa), ribonuclease A (14 kDa), and aprotinin (6 kDa).

*Characterization of purified MNCF:*
*Electrophoretic analysis.* SDS-PAGE was performed on 10% polyacrylamide gels under dissociating or reducing conditions.[13] The D-gal+ and D-gal− fractions of both crude MNCF and purified MNCF obtained from $3.6 \times 10^8$ peritoneal macrophages were applied and run for approximately 3 h at a constant current of 40 mA. The gel was silver stained.[14] The apparent MW of the proteins was determined from the calibration line obtained from the migration coefficient of known proteins which included rabbit IgG (150 kDa), phosphorylase-B (97 kDa), BSA (66 kDa), chicken egg albumin (45 kDa), chymotrypsinogen (25 kDa) and cytochrome C (12 kDa).

*Chromatofocusing.* Chromatofocusing[15] was performed on a MONO-P HR 5/5 column (Pharmacia LKB Biotechnology, Uppsala, Sweden) equilibrated with 0.025 M bis-Tris buffer, pH 7.1, operated at 20°C with a flow rate of 0.5 ml/min. The sample applied (1 ml) was the isolated MNCF and 1 ml fractions were collected. After elution with the initial buffer, the retained material was eluted with a pH gradient from 6 to 4 obtained using polybuffer 96 (Pharmacia LKB Biotechnology, Uppsala, Sweden). The material retained after the application of the pH gradient was then eluted with 1 M NaCl.

*Determination of biological activity:*

In vivo *migration assays.* MNCF activity was assayed by its ability to induce neutrophil migration into the peritoneal cavity or air pouch of rats pretreated with a glucocorticoid (0.5 mg of dexamethasone acetate ester/kg, subcutaneously; Merck, Darmstadt, Germany). Each sample was tested in groups of five to six rats. Air pouches were produced on the dorsum of rats as described by Edwards *et al.*[16] One h after dexamethasone, 1 or 3 ml samples were injected into the air pouch or peritoneal cavity, respectively. The animals were sacrificed by cervical dislocation 4 or 6 h later for the peritoneal or air pouch tests, respectively. The cells were immediately harvested by washing the respective cavities with 5 or 10 ml PBS containing albumin (0.1% w/v) and heparin (5 IU/ml). Total counts of harvested cells were performed in a Neubauer chamber. Differential counts were made on smears stained using Rosenfeld's panchromic method. The results are reported as the mean number (± S.E.M.) of neutrophils per ml of cavity wash.

In vitro *neutrophil chemotaxis migration assay.* Assays of *in vitro* neutrophil migration were performed as described by Bignold[17] in a 48-well chemotactic microchamber (Neuroprobe, Cabin John, MD). Human neutrophils were isolated from the heparinized peripheral blood of healthy human volunteers using mono poly resolving medium (Flow laboratories, Rockville, MD). After washing with RPMI 1640 medium, the neutrophils were resuspended in RPMI 1640 medium contained 0.1% (w/v) BSA (RPMI–BSA), to provide $10^6$ cells/ml. Typical preparations contained more than 95% viable neutrophils. Purified neutrophils were placed in the upper chamber while the lower chamber contained the test samples dissolved in RPMI–BSA. Random migration was assessed by using RPMI–BSA in the lower chamber. The peptide FMLP ($10^{-7}$ M) was used as the reference chemoattractant. The number of cells that migrated through the entire thickness of a 5 μm polycarbonate filter (Millipore Corp., Bedford, MA) during the 1 h incubation at 37°C in a 5% $CO_2$ atmosphere was counted. Five fields were counted for each assay and each sample was assayed in triplicate. The results are reported as the mean number (± S.E.M.) of neutrophils per field. The samples tested were crude MNCF as well as the various fractions obtained during the isolation of MNCF.

### Results

*Standardization of the conditions for the production and detection of MNCF:* We have confirmed our previous results demonstrating that LPS-stimulated macrophages release MNCF which induces neutrophil migration *in vitro* and in the peritoneal or air pouch cavities of dexamethasone-pretreated rats in a dose-dependent manner.[12] No MNCF activity was seen in LPS-stimulated macrophage monolayers incubated at 4°C rather than at 37°C. The number of neutrophils present in the peritoneal cavity of animals injected with this supernatant was similar to that observed after injection of PBS. For convenience, PBS was used as a negative control in subsequent experiments. The ability of MNCF to induce neutrophil migration after dexamethasone treatment was used to detect MNCF activity.

*The* D*-galactose binding property of MNCF and isolation:* Fig. 1 shows that most of the MNCF activity was not retained on the D-mannose (D-man+) affinity resin while the reverse was true for the D-galactose resin (D-gal+). Both this latter fraction and the fraction which did not bind the D-galactose (D-gal−) resin induced neutrophil migration *in vitro* (Fig. 2).

An electrophoretic analysis of the D-gal+ fraction showed four protein bands of 39, 45, 54 and 68 kDa (Fig. 3, lane b). In contrast, multiple bands were detected in the D-gal− fraction (Fig. 3, lane a).

The D-gal+ preparation was chromatographed on a Superdex 75 column and the ability of each fraction to induce neutrophil migration was initially assayed *in vitro.* A single eluate peak of chemotactic activity was detected in the material

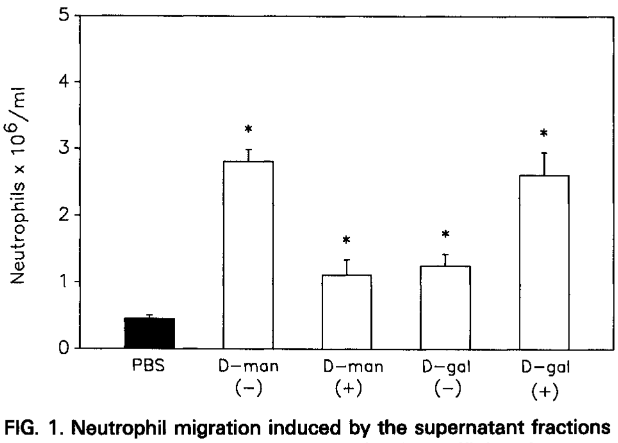

FIG. 1. Neutrophil migration induced by the supernatant fractions of LPS-stimulated macrophages submitted to affinity chromatography on immobilized sugar resins. The supernatant from 3.6 × $10^8$ LPS-stimulated macrophages was applied to agarose-D-galactose or agarose-D-mannose columns. The retained (D-gal+ and D-man+) or non-retained (D-gal− and D-man−) fractions were injected into peritoneal cavity of dexamethasone-pretreated rats (0.5 mg/kg, s.c., 1 h previously). The sample injected into each rat was obtained from 7.2 × $10^6$ macrophages. PBS was also injected. Neutrophil migration was evaluated after 4 h. The results are the mean number (± S.E.M.) of neutrophils per ml of peritoneal wash ($n$ = six rats/group). *$p < 0.05$ compared with the negative control (PBS); Student's $t$-test.

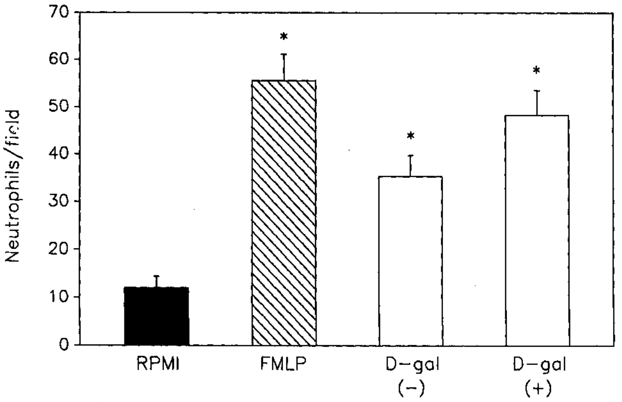

FIG. 2. Neutrophil chemotaxis *in vitro* induced by the D-gal+ and D-gal− fractions. The chemotactic activity of the D-gal+ and D-gal− fractions (from 4.5 × $10^6$ macrophages/well) and of FMLP ($10^{-7}$ M) was tested in a 48-well microchamber. Purified human neutrophils ($10^6$) were used per well and the assay was carried out in triplicate. Migration was evaluated after 1 h. The results are the mean number (± S.E.M.) of neutrophils per field (15 field). Basal chemotaxis was determined with RPMI 1640. *$p < 0.05$ compared to basal chemotaxis; Student's *t*-test.

eluted in the volume range of 9–11 ml, with maximal activity present in the fraction eluted in at 10 ml (Fig. 4A). Given these results, the fractions in the eluted volume range of 8–13 ml (14 to 90 kDa) were also assayed *in vivo* in dexamethasone-pretreated rats. MNCF activity was detected only in the fraction eluted at 10 ml, corresponding to an apparent MW of 54 kDa (Fig. 4B). Electrophoretic analysis of the active fraction revealed a single protein band with a position corresponding to 54 kDa (Fig. 3, lane c).

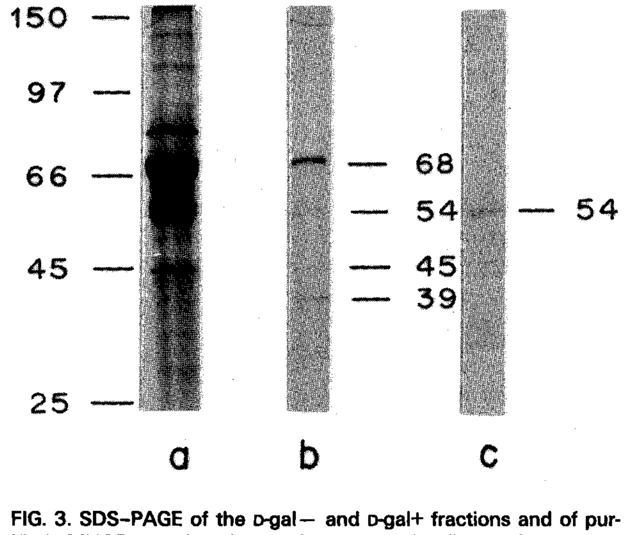

FIG. 3. SDS-PAGE of the D-gal− and D-gal+ fractions and of purified MNCF. D-gal− (lane a) and D-gal+ (lane b) fractions obtained from 3.6 × $10^7$ macrophages were applied to a 10% gel in sample buffer not containing mercaptoethanol. The purified MNCF (lane c) was obtained from 3.6 × $10^8$ macrophages. The gel was silver stained. The molecular weight markers were rabbit IgG (150 kDa), phosphorylase B (97 kDa), bovine serum albumin (66 kDa), chicken egg albumin (45 kDa), and chymotrypsinogen (25 kDa).

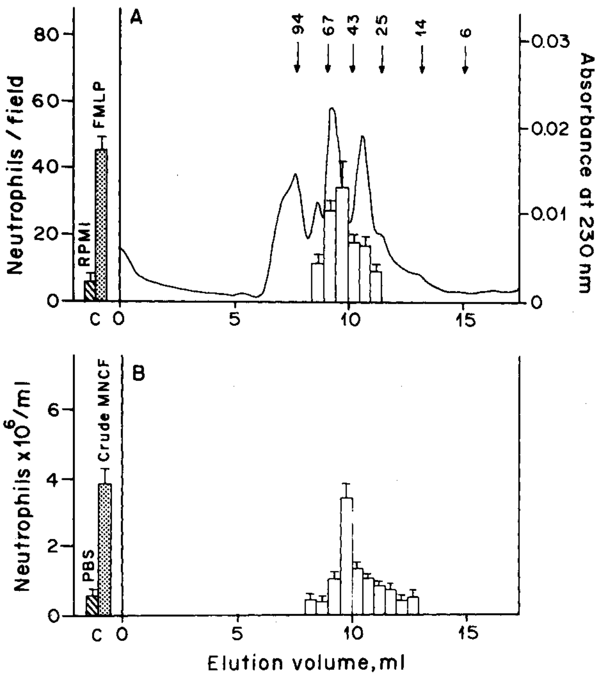

FIG. 4. Neutrophil migration induced *in vivo* and *in vitro* by fractions of D-gal+ material filtered on a Superdex 75 HR 10/30 column. The D-gal+ material (0.2 ml) obtained from 3.6 × $10^8$ macrophages was applied to a column equilibrated with PBS and operated at 20°C at a flow rate of 0.5 ml/min. Fractions of 0.5 ml were collected, ultradiafiltered on a YM-10 membrane (Amicon) and assayed *in vivo* and *in vitro* for the induction of neutrophil migration. Panel A: The *in vitro* chemotactic activity of the chromatographic fractions (from 9 × $10^6$ macrophages/well) and FMLP ($10^{-7}$ M) was tested in a 48-well microchamber as described in Fig. 2. The first bar represents the random migration induced by RPMI 1640. All fractions were tested but only neutrophil migration greater than the random migration is shown. The chromatographic profile shows the absorbance at 230 nm. Panel B: The *in vivo* neutrophil migration into the peritoneal cavity of dexamethasone-pretreated rats (0.5 mg/kg) induced by the gel filtration fractions eluted in the volume range corresponding to proteins of 14–90 kDa. The material injected per rat was obtained from 1.4 × $10^7$ macrophages. Neutrophil migration was evaluated as described in Fig. 1. The elution positions of proteins of known molecular weight are indicated by the arrows.

In the presence of 2-mercaptoethanol, only one protein band was seen upon electrophoresis, thus indicating that the MNCF is a single chain polypeptide (data not shown).

In the present study no attempt was made to calculate the recoveries and specific activities of the MNCF because of the scarcity of the material necessary for quantification of the biological activity and protein measurement. However, a rough estimation can be made by considering the number of macrophages necessary to induce a migration of 2–3 × $10^6$ neutrophil/ml of peritoneal wash in the dexamethasone-pretreated animals. In the majority of the MNCF preparations, 20–25% of the neutrophil chemotactic activity was recovered at each step of purification.

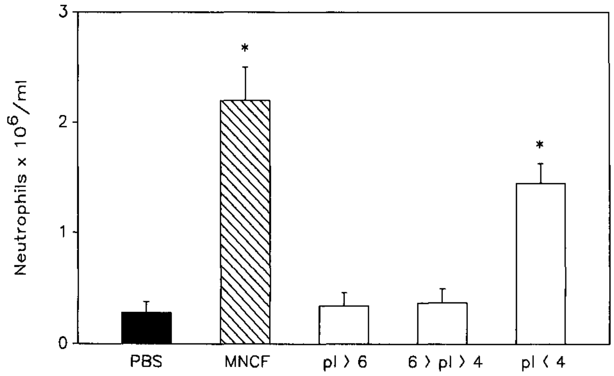

FIG. 5. *In vivo* neutrophil migration induced by fractions obtained by the chromatofocusing of purified MNCF. The fraction with *in vivo* activity from the Superdex 75 HR chromatography (from $3.6 \times 10^8$ macrophages) was applied to a Mono-P H 5/5 column equilibrated with L-histidine buffer (0.025 M, pH 6.2) and operated at 20°C at a flow rate of 0.5 ml/min. The elution was performed with polybuffer 96 which permits a pH gradient of 6 to 4. One ml fractions were collected and pooled: Pool I corresponds to fractions containing material with a pI greater than 6 (flow through), Pool II corresponds to material with a pI of 6–4 eluted on the pH gradient, and Pool III corresponds to material with a pI less than 4, eluted with 1 M NaCl. The pools were ultradiafiltered using a YM-10 membrane and assayed for their ability to induce neutrophil migration into the peritoneal cavities of dexamethasone-pretreated rats (material from $1.4 \times 10^7$ macrophages/rat). PBS was also assayed. The neutrophil migration induced by purified MNCF was also determined. In all cases, the migration was evaluated 4 h after the stimulus. The results are the mean number ($\pm$ S.E.M.) of neutrophils per ml of peritoneal wash ($n$ = six rats/group). $^*p < 0.05$ compared with negative control (PBS).

*Characterization of the acidic character of MNCF:* After chromatofocusing of purified MNCF, biological activity was recovered in the eluate corresponding to a protein with a pI less than 4.0 (Fig. 5). The acidic nature of the MNCF was confirmed by isoelectrofocussing which revealed a single band whose position also corresponded to an isoelectric point of 4.0.

### Discussion

We have described here a simple two-step purification process involving adsorption to a D-galactose column followed by gel filtration on Superdex 75, in order to obtain a homogeneous fraction MNCF (macrophage-derived neutrophil chemotactic factor) from the supernatant of LPS-stimulated macrophage monolayers. By using immobilized sugar resins and testing the MNCF activity *in vivo* and *in vitro*, we found that the component responsible for this activity was adsorbed to on a D-galactose but not a D-mannose column. SDS–PAGE analysis of the active D-galactose-bound fraction (D-gal+) showed four bands, corresponding to 39, 45, 54 and 68 kDa. When the D-gal+ preparation was filtered on Superdex 75, the *in vitro* and *in vivo* biological activity of MNCF was present in the fraction eluted in the volume corresponding to a protein of 54 kDa. SDS–PAGE analysis of this fraction also showed a single band corresponding to 54 kDa, a result which was unaltered under reducing conditions. The acidic nature of this protein (pI < 4) was demonstrated by chromatofocusing.

Recently, Ii *et al.*[18] cloned and described the primary structure of a macrophage asialoglycoprotein-binding protein (M-ASGP-BP). This 42 kDa lectin binds to galactose or *N*-acetylgalactosamine and belongs to a family of C-type animal lectins which show a high degree of homology with the 54 kDa hepatic asialoglycoprotein receptor.[18,19] It is possible that MNCF belongs to this family of C-type animal lectins or to the family of galectins.[20]

The homogeneous preparation of MNCF induced dexamethasone-resistant *in vivo* neutrophil migration which is in agreement with our previous observations with crude MNCF. There was an apparent discrepancy between the *in vitro* and *in vivo* chemotactic activity of the fraction which did not bind to D-galactose. This fraction stimulated migration *in vitro* but not our *in vivo* test (Fig. 1 and 2). The *in vitro* activity may be attributable to other chemotactic mediators expected to be present in the supernatant of endotoxin-stimulated macrophages. The chemotactic activities of these mediators were possibly not detected in the *in vivo* assay because the animals had been pretreated with dexamethasone. This is the case for IL-8,[9] $LTB_4$[21] and pro-inflammatory cytokines such as IL-1 and TNF.[11]

In conclusion, we have described the purification of a 54 kDa acidic protein, identified as macrophage-derived neutrophil chemotactic factor (MNCF). This protein causes *in vitro* chemotaxis as well as *in vivo* neutrophil migration in animals treated with dexamethasone.

### References

1. Ferreira SH. Are macrophages the body's alarm cells? *Agents Actions* 1980; **10:** 229–230.
2. Weir DM. Macrophage signal recognition. *Agents Actions* 1984; **15:** 2–11.
3. Unanue ER, Allen PM. The basis for the immunomodulation of macrophages and other accessory cells. *Science* 1987; **236:** 551–557.
4. Nathan CF. Secretory products of macrophages. *J Clin Invest* 1987; **79:** 319–326.
5. Stein M, Keshav S. The versatility of macrophages. *Clin Exp Allergy* 1992; **22:** 19–27.
6. Takemura R, Werb Z. Secretory products of macrophages and their physiological functions. *Am J Physiol* 1984; **246:** C1–C9.
7. Werb Z, Banda MJ, Takemura R, Gordon S. Secreted proteins of resting and activated macrophages. In: Weir A, ed. *Handbook of Experimental Immunology.* Oxford: Blackwell Sci. Publ; 1986; 367–397.
8. Adams DO, Hamilton JA. Macrophages as destructive cells in host defense. In: Gallin JI, Goldstein IM, Snyderman R, eds. *Inflammation: Basic Principles and Clinical Correlates.* New York: Raven Press; 1992; 637–662.

9. Ribeiro RA, Flores CA, Cunha FQ, Ferreira SH. IL-8 causes *in vitro* neutrophil migration by a cell-dependent mechanism. *Immunology* 1991; **73:** 472–477.
10. Rot A. Binding of neutrophil attractant/activation protein-1 (interleukin 8) to resident dermal cells. *Cytokine* 1992; **4:** 347–352.
11. Faccioli LH, Souza GEP, Cunha FQ, Poole S, Ferreira SH. Recombinant interleukin-1 and tumour necrosis factor induce neutrophil migration *in vivo* by indirect mechanisms. *Agents Actions* 1990; **30:** 344–349.
12. Cunha FQ, Ferreira SH. The release of a neutrophil chemotactic factor from peritoneal macrophages by endotoxin: inhibition of glucocorticoids. *Eur J Pharmacol* 1986; **129:** 65–76.
13. Laemmli UK. Cleavage of structural proteins during the assembly of the head of bacteriophage $T_4$. *Nature* 1970; **227:** 680–685.
14. Blum H, Beier H, Gross JH. Improved silver staining of plant proteins, RNA and DNA in polyacrylamide gels. *Electrophoresis* 1987; **8:** 93–99.
15. Sluyterman LA, Wijdenes J. Chromatofocusing: isoelectric focusing on ion exchange columns. I. General principles. *J Chromatogr* 1978; **150:** 31–35.
16. Edwards JCW, Sedgewick AD, Willoughby DA. The formation of structure with the features of synovial lining by subcutaneous injection of air. An *in vivo* tissue culture system. *J Pathol* 1981; **153:** 147–156.
17. Bignold LP. Kinetics of chemoattractant of polymorphonuclear leukocytes towards *N*-formyl peptide studied using a novel polycarbonate (Nuclepore) membrane in the Boyden Chamber. *Experientia* 1988; **44:** 518–521.
18. Ii M, Kurata H, Itoh N, Yamashina I, Kawasaki T. Molecular cloning and sequence analysis of cDNA encoding the macrophage lectin specific for galactose and *N*-acetylgalactosamine. *J Biol Chem* 1990; **256:** 11295–11298.
19. Drickamer K, Mamon JF, Binns G, Leung JO. Primary structure of the rat liver asialoglycoprotein receptor. *J Biol Chem* 1984; **259:** 770–778.
20. Barondes SH, Cooper DNW, Gitt MA, Leffler H. Galectins. Structure and function of a large family of animal lectins. *J Biol Chem* 1994; **269:** 20807–20810.
21. Oda T, Katori M. Inhibition site of dexamethasone on extravasation of polymorphonuclear leucocytes in the hamster cheek pouch microcirculation. *J Leucocyte Biol* 1992; **52:** 337–342.

ACKNOWLEDGEMENTS. This work was funded by the Fundação de Amparo à Pesquisa do Estado de São Paulo (FAPESP) and by the Conselho Nacional de Pesquisa e Desenvolvimento (CNPq), Brazil. The authors thank Mrs Sandra Maria Oliveira Thomaz, Maria Imaculada Bragheto, Neomésia Issajuara da Silva Freire and Sérgio Roberto Rosa for expert technical assistance.

**Received 31 March 1995; accepted 4 April 1995**
